# Development and evaluation of a remote training strategy for the implementation of mental health evidence-based practices in rural schools: pilot study protocol

**DOI:** 10.1186/s40814-022-01082-4

**Published:** 2022-06-17

**Authors:** Ricardo Eiraldi, Barry L. McCurdy, Muniya S. Khanna, Jessica Goldstein, Rachel Comly, Jennifer Francisco, Laura E. Rutherford, Tara Wilson, Kathryn Henson, Thomas Farmer, Abbas F. Jawad

**Affiliations:** 1 Roberts Center for Pediatric Research, Children’s Hospital of Philadelphia, 2716 South Street, Room 8293, Philadelphia, PA 19146-2305 USA; 2 Department of Pediatrics, University of Pennsylvania Perelman School of Medicine, Philadelphia, PA USA; 3 School of Professional and Applied Psychology, Philadelphia College of Osteopathic Medicine, Philadelphia, USA; 4 OCD and Anxiety Institute, Plymouth Meeting, PA USA; 5 Devereux Center for Effective Schools, King of Prussia, PA USA; 6 School of Education, University of Pittsburgh, Pittsburgh, PA USA

**Keywords:** Rural schools, User-centered design, Positive behavioral interventions and supports, Mental health evidence-based practices, Tier 2

## Abstract

**Background:**

An increasing number of schools in rural settings are implementing multi-tier positive behavioral interventions and supports (PBIS) to address school-climate problems. PBIS can be used to provide the framework for the implementation of evidence-based practices (EBPs) to address children’s mental health concerns. Given the large service disparities for children in rural areas, offering EBPs through PBIS can improve access and lead to better long-term outcomes. A key challenge is that school personnel need technical assistance in order to implement EBPs with fidelity and clinical effectiveness. Providing ongoing on-site support is not feasible or sustainable in the majority of rural schools, due to their remote physical location. For this reason, remote training technology has been recommended for providing technical assistance to behavioral health staff (BHS) in under-served rural communities.

**Objectives:**

The purpose of this study is to use the user-centered design, guided by an iterative process (*rapid prototyping*), to develop and evaluate the appropriateness, feasibility, acceptability, usability, and preliminary student outcomes of two online training strategies for the implementation of EBPs at PBIS Tier 2.

**Methods:**

The study will employ a pragmatic design comprised of a mixed-methods approach for the development of the training platform, and a hybrid type 2, pilot randomized controlled trial to examine the implementation and student outcomes of two training strategies: Remote Video vs. Remote Video plus Coaching.

**Discussion:**

There is a clear need for well-designed remote training studies focused on training in non-traditional settings. Given the lack of well-trained mental health professionals in rural settings and the stark disparities in access to services, the development and pilot-testing of a remote training strategy for BHS in under-served rural schools could have a significant public health impact.

**Ethics and dissemination:**

The project was reviewed and approved by the institutional review board. Results will be submitted to ClinicalTrials.gov and disseminated to community partners and participants, peer-reviewed journals, and academic conferences.

**Trial registration:**

ClinicialTrials.gov, NCT05034198 and NCT05039164

## Background

Eighty-four percent of Mental Health Professional Shortage areas in the USA are located in rural and frontier areas [1]. Children and adolescents in rural settings are less likely to receive services compared to their urban and suburban counterparts and even fewer are likely to receive evidence-based care [2, 3]. Schools have become more involved in the delivery of mental health services and hold great potential for increasing access for children and adolescents. Innovations in training and service delivery are needed to improve mental health care quality and availability in rural schools [4]. Evidence-based practices (EBPs) can be incorporated into school-wide multi-tiered systems that are currently being used to improve school climate and safety. School-wide positive behavioral interventions and supports (PBIS), a service-delivery framework based on the public health model, is one example [5, 6]. A growing number of schools in rural areas are employing PBIS [7–10]. Given the large service disparities for children in rural areas, offering EBPs through PBIS can improve access and lead to better long-term outcomes [11]. Our research team has used PBIS to incorporate EBPs at Tier 2 for children with, or at risk for, mental health disorders in urban schools [12–14]. We have demonstrated that school personnel, with or without prior mental health training, can implement Tier 2 interventions with fidelity and clinical effectiveness (i.e., child symptom improvement) if given adequate technical assistance (i.e., training support) [12, 13, 15, 16]. In urban and suburban schools, this training can be provided to school staff on site. However, providing on-site training is not feasible or sustainable in the majority of rural schools, due to their remote physical location. For this reason, remote training technology has been recommended for the training of behavioral health staff (BHS) in under-served rural communities [17, 18].

Remote training technology offers the potential to provide training for behavioral health staff in rural schools. Based on our reading of the relevant literature and our collective experience developing programs in the school setting, we propose that the development of a training strategy for BHS in rural school settings ought to (a) use a participatory design with school personnel, (b) employ web-based training technologies, (c) include a training system for BHS to enhance knowledge and skill needed for implementation, and (d) incorporate implementer and school context factors to increase perceived feasibility, appropriateness and acceptability by stakeholders.

We will involve school BHS in the development of the training strategy using the user-centered design approach [19] guided by an iterative development framework. The iterative framework, *rapid prototyping*, originally used for software development [20, 21], is based on a cyclic process of analyzing data from users in order to improve successive prototypes. Applied to this project, prototyping will involve the creation of “low fidelity” versions of the training platform that contains key functions of interest in order to test a concept, and facilitate rapid evaluation and feedback [19]. Following the evaluation of the early prototypes, a fully functional “high fidelity” prototype is created that is more similar to the final product and that offers fully interactive content [22]. Rigor is achieved in this process through the systematic, repetitive, and recursive nature of the qualitative and quantitative data analysis from user feedback.

Carefully considering the perspectives of BHS in the development of the training strategy might make it more likely that they will participate in the training and that they continue using EBPs with students in the future. User-centered design, also known as participatory design, is an approach to product development that has increasingly been used for the development of psychosocial interventions [19, 23]. We will work with stakeholders to ensure that the training strategy is easy to use and understand by school behavioral health staff, that is acceptable for the school context, and that is appropriate for their needs [24].

Advantages of web-based remote training include flexibility, accessibility, cost-efficiency, potential for both didactic and interactive learning, and consistency in quality [17, 25]. Remote online training allows for synchronous (i.e., interactive) supervision and feedback from a supervisor anywhere in the country. This allows for the trainee to be able to receive ongoing consultation or supervision on site without the time and cost of travel [25–27]. Advantages to training and consultation using an online strategy include the potential for (a) self-paced learning, (b) trainee competency and adherence checks, and importantly, (c) the resources/time benefits of 24-h and in-school/in-home access to learning and treatment materials.

Remote technology-enhanced programs have been found to be acceptable and feasible in community settings [28, 29]. For example, a study testing the effectiveness of consultation via video for improving teacher behavior management found that perceived acceptability of consultation by teachers increased from “acceptable” at baseline to “highly acceptable” at post-consultation [30]. Another study, conducted with teachers in rural schools, showed teleconsultation to be feasible, acceptable, and effective at improving teacher classroom behavior management [31]. A systematic review of studies using teleconsultation in schools showed teleconsultation to be an effective service delivery method [32].

Studies suggest that technology-based training methods, particularly when developed using the latest multimedia and interactive design formats, may be more effective than manuals alone and as effective as face-to-face training workshops in disseminating EBPs to community mental health professionals [17, 24, 29, 33]. Our online strategy differs in a number of ways from the consultation approach used in previous studies. We will offer protected access to asynchronous training materials such as training video “modules” that include didactic content, audio and visual examples, as well as treatment materials and resources, all of which can be viewed at the trainee’s individual pace and convenience. The study will also examine the potential added benefit of offering synchronous consultation by expert consultants to the training package.

Initial training workshops and ongoing consultation with BHS are key strategies for implementing EBPs in schools. Multicomponent training strategies for mental health therapists, comprised of an initial workshop followed by ongoing consultation, have been found to be more effective than a single workshop for enhancing therapist clinical skills and knowledge, treatment adherence, and clinical outcomes [34–36]. The literature has shown that an initial training workshop is a necessary training component. However, for the rural school context, it is not known whether providing additional interactive consultation would be necessary if BHS are instead provided with step-by-step instructions on how to implement EBPs via asynchronous video. With an asynchronous video strategy, BHS could also be provided intervention materials (e.g., intervention manuals) that can be downloaded on demand. Training via asynchronous video would be more feasible for busy BHS and potentially less expensive than attending pre-scheduled ongoing synchronous consultation. In this study, we will fill a void in the literature by examining the amount and type of resources needed by BHS in rural schools in order to implement mental health EBPs with fidelity and clinical effectiveness. Also, the results of the present study will inform the composition of the training strategy used in a future larger study in rural schools.

Proctor and colleagues propose that the perceived appropriateness, feasibility, and acceptability of a health innovation are key to its implementation success [37, 38]. Appropriateness refers to the perceived fit, relevance, or compatibility of the innovation for a specific setting [37, 39]. Feasibility refers to the extent to which an innovation can be successfully used in a particular setting [37, 39]. Acceptability refers to the perception among EBP implementers as to whether the innovation is agreeable, palatable, or satisfactory [37, 39]. A nested confirmatory factor analysis provided evidence of structural validity for measuring these constructs, with the three-factor model (appropriateness, feasibility, acceptability) yielding acceptable model fit and high-scale reliability [40]. We will use these measures in the study. We will also measure usability of the training strategy [41]. Usability, which is defined as the degree to which a program can be used easily, efficiently, with satisfaction, and low user burden by a particular stakeholder [42], is a key outcome of user-centered design [19]. Of particular importance for this study is that appropriateness, feasibility, acceptability, and usability are mutable factors that can be used in an iterative manner with key stakeholders to guide the development and refinement of a health innovation [37, 43]. We will measure these four constructs to guide the development and implementation of the remote training strategies.

### Development and evaluation of remote training strategies

In the current pilot study, we will develop, revise, and evaluate asynchronous video modules for use in rural schools. Following the development of the training modules, we will conduct a pilot study to examine implementation and child outcomes of two training strategies for BHS: (a) initial training workshop followed by asynchronous didactic video training (Remote Video) and (b) initial training workshop followed by asynchronous didactic video plus synchronous video coaching (Remote Video plus Coaching). At the conclusion of the study, we will submit a fully powered, Hybrid Type 3 R01 grant proposal to examine implementation outcomes (adoption, penetration, fidelity, cost) of the remote training platform with a larger sample of rural schools.

### Objectives/aims

The primary aims of the study are:

- To obtain input from school stakeholders about barriers and facilitators of remote online training by employing a user-centered research approach
- To use user-centered design guided by an iterative rapid prototyping approach to develop asynchronous video modules based on preliminary studies and aim 1 data
- To conduct a pilot trial of Remote Video vs. Remote Video plus Coaching

## Method

The present protocol has been registered within ClinicalTrials.gov (registration numbers NCT05034198 and NCT05039164). The final study report will be prepared in accordance with the reporting guidance provided in the CONSORT extension for reporting pilot randomized controlled trials.

### Design

The study will employ a pragmatic design comprised of a mixed-method approach for aims 1 and 2 and a 2-arm, pilot randomized controlled trial, with a type 2 hybrid design [44] for aim 3. Aims 1 and 2 will be completed during years 1 and 2 and aim 3 during years 3–5 of the study.

### Randomization

We will invite 100 schools to participate and we estimate that approximately 30 schools (30%) will agree to participate in the initial interview with BHS (aim 1). The 30 participating schools will be included in the training strategy development (aAim 2). We will assign 16 schools to participate in the hybrid pilot trial (aim 3). After receiving school consent to participate, schools will be stratified based on geographic location and a computer-generated randomization list will be prepared to randomize the 16 schools in a 1:1 ratio to either Remote Video or Remote Video plus Consultation (8 schools/arm).

### Study flowchart

**Fig. 1:**
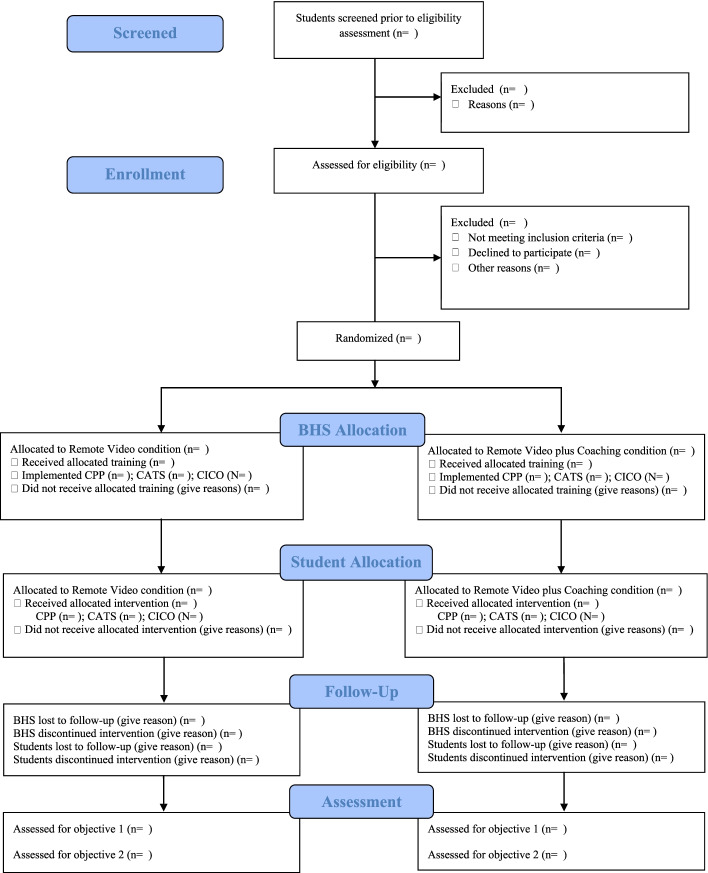
Study flowchart

Figure 1 illustrates the study flowchart.

### Inclusion criteria

Any rural school, designated by the US Census Bureau, with a PBIS program that is implementing Tier 1 with fidelity, with or without a functioning Tier 2. Implementing Tier 1 with fidelity is required because Tier 1 is foundational for the development of mental health interventions at the advanced tiers of support [45]. Any BHS (e.g., school counselor, school social worker) or teacher, with or without experience implementing Tier 2 interventions, based at a school implementing PBIS, would be eligible for inclusion in the study.

For aim 3, inclusion of students to receive a Tier 2 intervention is as follows:

- Attending one of the participating schools
- Being in grades 4–8
- Identified by the Tier 2 team as not responding to Tier 1 intervention, thus needing Tier 2 support
- Scoring > 1 SD above the mean on the Emotional Symptoms or Conduct Problems scales of the Strengths and Difficulties Questionnaire (SDQ) [46] completed by a parent or a teacher

### Exclusion criteria

School staff from schools not implementing PBIS will not be included in the study, nor will students who do not meet inclusion criteria. Students with a history of intellectual disability or serious developmental delays according to school records will not be included.

### Measures

Participant burden for parents and teachers is minimal. BHS will be asked to complete more measures (see Table 1) than parents and teachers, but measures are typically brief. Measures that require more time (e.g., qualitative interviews) are used less often. We will use REDCap, secure email, and regular mail for data collection. Qualitative interviews will be conducted over the phone.

**Table 1:** Measures by variable/construct, measure characteristics, timepoint, method, informant, and time burden

Variable/construct	Measure	Measure characteristics	Timepoint	Method	Informant	Time
Aim 1						
Barriers and facilitators	Interview guide # 1	A semi-structured qualitative interview will be conducted with BHS to elicit views about perceived barriers and facilitators to participation in consultation sessions and conducting groups with students (e.g., *What would make it difficult for you to participate in consultation sessions and conduct groups with students? Now, please tell me what would make it easier for you to participate in remote training, receive consultation remotely or conduct groups with students?*)	Pre-trial	Coding	Behavioral health staff	20 min
Aim 2						
Assess prototype	Interview guide # 2	The interview includes a description of the first platform prototype; it will describe each training and consultation component. BHS will be asked whether the different components of the training and consultation and group implementation would be feasible (e.g., *We’re interested in your thoughts about how feasible it would be to use remote video technology in your school*) and acceptable (e.g., *We’re interested in your thoughts about how acceptable the remote video technology is*) using a 5-point scale [46]. They will also be asked why the component is or is not feasible/acceptable; whether they would be willing to participate in remote consultation.	Pre-trial	Coding	Behavioral health staff	30 min
Assess prototypes	Surveys # 1–3	The surveys include the Intervention Appropriateness Measure [IAM], the Acceptability of Intervention Measure [AIM], and the Feasibility of Intervention Measure [FIM] [40]. The measures are comprised of 4 items, each rated on a 5-point scale (1=completely disagree to 5=completely agree). Scale refinement based on measure-specific CFAs and Cronbach alphas using vignette data produced 4-item scales (*α*’s from 0.85 to 0.91). A three-factor CFA exhibited acceptable fit (CFI = 0.96, RMSEA = 0.08) and high factor loadings (0.75 to 0.89), indicating structural validity. ANOVA showed significant main effects, indicating known-groups validity. Test-retest reliability coefficients ranged from 0.73 to 0.88. Regression analysis indicated each measure was sensitive to change in both directions [40]. Survey # 3 will also include the Intervention Usability Scale (IUS), an adaptation of the System Usability Scale (SUS) [41]. The SUS is a widely used scale for assessing usability of digital products. We adapted the SUS for the evaluation of instructional videos. Respondents will also be asked to provide comments to explain their answers (e.g., “*please comment on the video about using remote consultation technology*”).	Pre-trial	Rating scale and coding	Behavioral health staff	30 min
Pre-trial activities						
Tier 2 screening	Strengths and Difficulties Questionnaire (SDQ) [46] with Impact Supplement [47]	The SDQ is a 25-item, 3-point scale (0 = not true; 2 = certainly true) questionnaire used to assess the psychological adjustment of children and youth, ages 4–17.	Pre-treatment	Rating scale	Parents/teachers	5 min
Aim 3: implementation trial						
Implementation measures						
Content fidelity of group CBT	Coping Power and CATS Content Fidelity Checklist (CFC) [48]	The CFC reflects each activity component of the session agenda of the treatment protocols. Raters use a yes/no response scale to indicate whether or not the implementer covered a particular component as captured in audio recordings of the group sessions.	Ongoing	Coding	Research staff	40 min
Content fidelity of CI/CO	Check-In/Check-Out Fidelity Checklist [49–51]	The Check-In/Check-Out Fidelity Checklist is a 9-10-item checklist used by Tier 2 implementers during morning check-in and afternoon check-out rated as either occurring or not occurring.	Weekly	Coding	Research staff	10 min
Adoption	Adoption Inventory (AI)	The AI is an Excel track sheet listing the number of times each intervention is used per school, per condition	Ongoing	Coding	Research staff	1 min
Dosage	Dosage Inventory (DI)	The DI is an Excel track sheet exported from the project website listing the number of times and length of time each video module is accessed by BHS in each condition	Ongoing	Digital	Research staff	5 min
Penetration	Penetration Inventory (PI)	The PI is an Excel track sheet listing EBP penetration at the student level (students receiving EBPs at Tier 2)	Ongoing	Coding	Research staff	1 min
Student outcome measures						
Mental health symptoms	Behavior Assessment System for Children - 3^rd^ Edition (BASC-3) [52]	Parents will complete either the web-based or paper and pencil version of the BASC-3. The BASC-3 is a 138-item, 4-point, Likert-type (1=never, 2=sometimes, 3=often, 4=almost always) rating scale for assessing parental report of child mental health functioning, standardized for ages 2.5 to 18 years. The BASC-3 has excellent psychometric properties. The BASC-3 (*Aggression, Conduct Problems,* and *Anxiety* scales) will be used for children being considered for Tier 2 and administered at pre- and post-treatment for children who participate in CPP, CATS, or CICO.	Pre/post-treatment	Rating scale	Parent	20 min
Student academic engagement	Engagement versus Disaffection with Learning - Teacher Report (EvsD-Teacher) [53, 54].	The EvsD will be completed by teachers for all students receiving Tier 2 interventions. This is a 20-item, four-point (1 = not at all true; 4 = very true) instrument with four sub-scales: (a) *Behavioral Engagement*, (b) *Emotional Engagement*, (c) *Behavioral Disaffection*, and (d) *Emotional Disaffection*. Internal consistency for students in grades 3–6 was .81–.87 for the four subscales. We will use the average score for each of the four scales at pre- and post-participation in CPP, CATS, or CICO.	Pre/Post-treatment	Rating scale	Teacher	10 min
Perception of training support	Qualitative Interview Guide # 3	Semi-structured qualitative interviews are conducted with Tier 2 implementers and administrators in each condition to elicit views and perspectives about the perceived feasibility, acceptability, appropriateness, and usability of the training support they received.	Post-trial	Coding	Behavioral health staff/administrators	30 min

### Tier 2 interventions

None of the schools will have any significant prior experience implementing mental health EBPs at Tier 2. Research consultants, supervised by licensed clinical psychologists, will provide technical assistance support to members of the Tier 2 team (i.e., BHS). In previous studies conducted by our team [16, 48, 55], school personnel expressed a desire to receive technical assistance for the implementation of EBPs for the most common mental health difficulties. As such, we will support BHS as they implement interventions for externalizing and anxiety problems, which are among the most common mental health problems in schools [56]. The three EBPs that schools will use during the pilot trial are the Coping Power Program (CPP) [57] for externalizing behavior disorders, CBT for Anxiety Treatment in Schools (CATS) [58] for anxiety disorders, and Check-in/Check-out (CICO) [59] for externalizing disorders. CPP and CATS will be implemented in a group format during a lunch period with students of similar developmental level (e.g., 4th and 5th or 7th and 8th grade together). We limit participation to students in grades 4–8 because the group EBPs are appropriate for this age group. Tier 2 implementers could opt to use CICO for individual students who present with externalizing problems.

The CPP intervention consists of twelve 45-min sessions. It teaches anger management skills, perspective taking, and problem solving. This intervention has been found to be effective at reducing aggressive behavior, covert delinquent behavior, and substance abuse among aggressive boys, with gains maintained at 1-year follow-up [60]. Growth curve analyses showed that CPP had linear effects for 3 years after intervention on reductions in aggressive behavior and academic behavior problems [61].

The CATS intervention is an adaptation of Friends for Life (FRIENDS) [62]. It teaches children how to recognize feelings of anxiety and physical reactions to anxiety, clarify thoughts and feelings in anxiety-provoking situations, develop a coping plan, evaluate their own performance, and provide self-reinforcement. The adapted protocol retains the core elements of evidence-based CBT for anxiety and the FRIENDS group format. Adaptation decisions for FRIENDS were based on our collective experience with the protocol, two previous implementation studies [13, 15], and focus groups and qualitative interviews with stakeholders. The adapted intervention is a briefer (8-session) and more feasible, engaging, and relevant for students in under-resourced schools than the original FRIENDS.

The CICO intervention is a targeted, individually administered, Tier 2 intervention for students at risk of developing externalizing mental health disorders [63]. The CICO intervention is designed to provide immediate feedback (i.e., at the end of each class period) to students, based on the use of a daily report card. This feedback is developmentally sensitive [63]. Implementers meet individually with students for a brief “check-in” in the morning and a brief “check-out” in the afternoon. Research on the use of CICO has shown it to be effective in reducing externalizing problems with elementary school students [63, 64]. The CICO intervention will be offered to individual students for a variable length of time, depending on need. Each school will be instructed to select CICO and one of the two CBT protocols for use during the pilot trial.

### Training strategy development

**Fig. 2:**
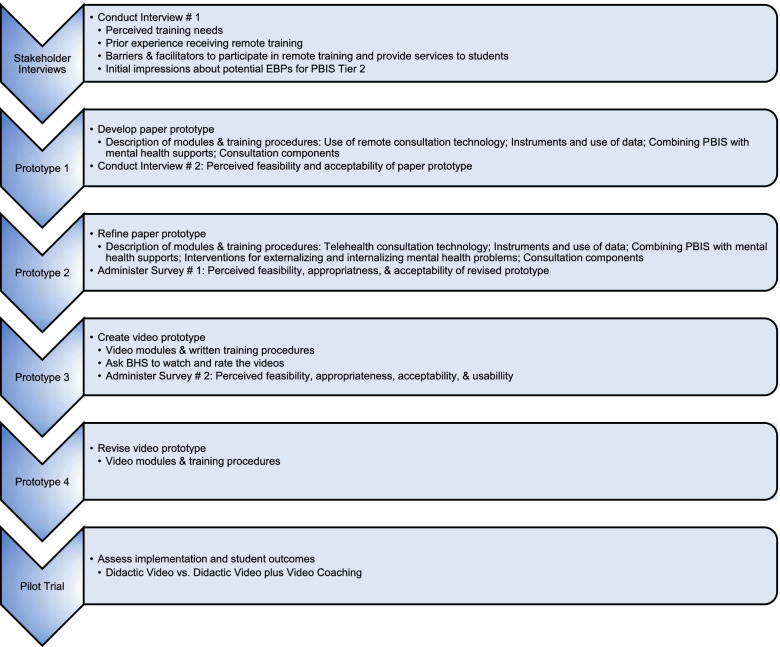
Development and evaluation of remote training platform

We will use evaluative and iterative strategies [65] to ensure that the remote training strategy is a good fit with the rural school context. Given that the training strategies will be used in schools with specific culture and administrative requirements, and by BHS who might have opinions and attitudes about receiving remote training and consultation, we will use a participatory approach to assess barriers and facilitators to participation in remote training (see Fig. 2).

### Aim 1: Initial stakeholder input

Thirty BHS (school counselors or school social workers; one per school) will participate in a semi-structured interview (interview guide # 1) of perceived barriers to and facilitators of participation in consultation sessions and conducting groups with students (e.g., *What would make it difficult for you to participate in consultation sessions and conduct interventions with students? Now, please tell me what would make it easier for you to participate in remote training, receive consultation remotely or conduct groups with students?*)

### Aim 2: Remote training strategy development

After analyzing the results of the first wave of interviews, we will use a second semi-structured interview containing descriptions of training modules, consultation components, and potential EBPs and ask the same 30 BHS to evaluate them (BHS interview guide # 2). The second semi-structured interview will include a description of the first remote training prototype; it will describe each training and consultation component, a rationale for the need for each component, a description of EBPs that will be offered (e.g., CPP) and procedures (e.g., steps needed to implement the component) and approximate time required for training modules, consultation sessions, and intervention implementation. Participants will be asked to evaluate, using a 5-point scale, the feasibility and acceptability of different components of the training and consultation and intervention implementation. They will also be asked why the component is or is not feasible/acceptable [66] and whether they would be willing to participate in remote consultation. After analyzing the second set of interviews, we will revise the description of the remote training strategy and ask the 30 BHS to complete three brief questionnaires about the perceived appropriateness, feasibility, and acceptability [40] of the revised, second prototype (BHS survey # 1).

Following the stakeholder’s evaluation of the second prototype, we will develop the actual training modules (third prototype). These will be a set of asynchronous (non-interactive) training videos. The development of the modules will be based on the training literature, our preliminary studies, and evaluation of the previous prototypes.

### Asynchronous video components

Mental health trainers with expertise in the treatment of externalizing and internalizing behavior disorders will video-record the training modules and produce them using lecture capture technology (i.e., showing speaker and PowerPoint slides on a split screen). When appropriate, training modules will include both didactic and active learning activities such as role-plays and behavior rehearsals by project staff, showing select sections of video-recorded sessions with students, and demonstration of techniques [35, 67].

Video modules will address both (a) specific interventions (i.e., CPP, CICO, CATS) and (b) general support for the implementation of EBPs. Modules about specific interventions will include a brief discussion of the theoretical background of the particular EBP, its development (theoretical rationale, key components, efficacy/effectiveness findings), and a detailed review of the group sessions (content, structure, process, implementation challenges). General modules might include (a) use of remote consultation technology; (b) description of consultation procedures; (c) instruments and use of data; (d) incorporating EBPs into PBIS [68]; (e) screening; (f) group behavior management; and (g) implementation barriers. Some videos (e.g., instruments and use of data) will be relatively brief, while other videos (e.g., CATS) will be longer in order to provide step-by-step instruction on how to implement the intervention.

### Video evaluations by school behavioral health staff

The 30 BHS from aim 1 will be asked to review and evaluate the asynchronous video modules by connecting to a project website. Immediately after BHS watch the videos, they will be asked to complete four brief surveys regarding the appropriateness, feasibility, acceptability [40], and usability [19] of each training module and provide comments about each (e.g., *Please comment on the video about using the remote consultation technology. What worked? What did not work? What changes do you suggest?*) (BHS survey # 2). Following a review of the questionnaires, further revisions will be made to the training modules (e.g., videos, manuals) and consultation procedures (fourth prototype).

If, at the end of the second mini-trial, we conclude that the training and procedures are not yet ready for the pilot trial, an additional iteration of the training strategy will be conducted in two additional schools following procedures similar to those described above. If no further iterations are necessary, we will proceed to the randomized pilot trial.

### Aim 3: Hybrid type 2 pilot trial

**Fig. 3:**
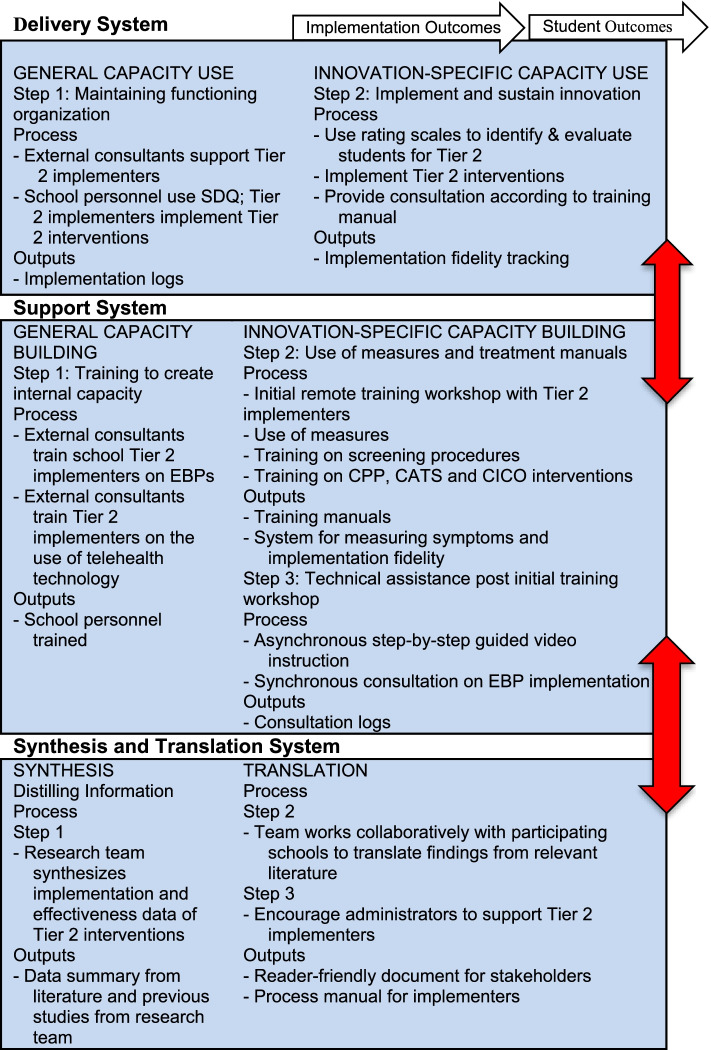
Interactive systems framework for dissemination and implementation

All activities related to the training of school personnel and implementation of EBPs for aim 3 are guided by the Interactive System Framework for Dissemination & Implementation (ISF) [69] (see Fig. 3). ISF is intended to be a “heuristic for understanding key systems, key functions, and key relationships relevant to the dissemination and implementation process” (p. 179) [69]. ISF is composed of three interrelated systems: Synthesis and Translation System (STS), Support System (SS), and Delivery System (DS). The function of STS is to distill information innovations and prepare them for implementation by service providers. SS supports the work of those who put the innovation into practice. The primary function of DS is the implementation of innovations in “real world” settings [69, 70]. We will use all three systems because they provide a roadmap for distilling information about the implementation of EBPs in schools, training of school personnel, and implementation of EBPs by school personnel.

We will conduct the pilot trial in 16 schools (8 per arm). It is estimated that a total of 48 behavioral health staff (3 staff per school — 24 per arm) and 208 students (13 students per school — 104 per arm) will participate in this pilot study. We expect that each school will have one staff member with prior mental health training (e.g., school counselor). This person will be expected to implement one of the group-EBPs. The two other staff members will be tasked with implementing the individualized CICO intervention, as this intervention can be implemented by school staff without prior mental health training [49, 51]. We estimate that each BHS will conduct one CPP or CATS group with 5–6 students of similar developmental level (e.g., a group of 4th and 5th graders; 40 students total) and that each school staff in CICO will implement the intervention with 4 individual students of any school grade (64 students total).

### Study conditions

The study conditions will be (a) Remote Video and (b) Remote Video plus Coaching. School staff assigned to Remote Video will participate in an initial synchronous training workshop followed by asynchronous video training. They will also be given copies of the intervention manuals and other related material. School staff assigned to Remote Video plus Coaching will participate in an initial synchronous training workshop followed by asynchronous video plus synchronous coaching via Webex.

#### Initial training

Research consultants will conduct a synchronous training workshop in August of each year via a video-conferencing platform (Webex) for all school personnel involved in CATS, CPP, and CICO. Tier 2 implementers will be instructed on the use of data to identify and assign students at risk for behavioral and emotional disorders into Tier 2. The Tier 2 implementers will also be taught a competency framework for mental health and PBIS [71, 72], strategies for enhancing school personnel knowledge of mental health “warning signs” among students through in-service training, and how to access the online materials. The training related to “warning signs” will be conducted in order to help teachers identify students who could benefit from the interventions.

Tier 2 implementers will be instructed to use training manuals and adherence checklists for Tier 2. The Tier 2 team will be trained on the use of a mental health screening instrument (SDQ) [47] and a multi-axial parent rating scale (Behavioral Assessment System for Children, Third Edition (BASC-3) [52]) and other instruments used in the study. Implementers will be introduced to a competency model for CBT [73]. They will also learn about how to deal with implementation barriers (e.g., scheduling sessions, conducting exposure tasks) [74]. Training content and procedures will be based on adult learning characteristics (e.g., propensity to learn from experience, capacity to reflect on performance and apply knowledge, and self-motivation) [33, 75].

#### Guided video

Following the initial training workshop and after implementers have identified students for interventions, Tier 2 implementers will be given access to the training videos developed for aim 2. The videos will be made available through a website that has the ability to measure how many times each video has been accessed by the Tier 2 implementer and for how long. Each participant will be given access to the videos that correspond to the interventions that they plan to use. All Tier 2 implementers will be given access to videos that address general topics (e.g., how to use data to identify students for participation; dealing with implementation barriers).

#### Coaching

Research team consultants will provide synchronous consultation to BHS using Webex. The consultation will have two main components: (a) session preparation (CPP/CATS) or review and planning (CICO) and (b) coaching.

Session preparation for CPP and CATS will consist of (a) discussing referrals to the groups; (b) conducting a step-by-step walkthrough of the session objectives; (c) reviewing the CBT principles behind the treatment components for that session; (d) encouraging adherence and the use of active learning strategies; (e) problem-solving barriers to implementation and helping BHS reflect on past challenges (e.g., attendance problems, organizational barriers, materials/resources) in order to successfully implement the next session with appropriate adaptations as needed; and (f) enhancing BHS’s use of empathy and positive reinforcement through modeling. These procedures have been successfully used by our team in previous school-based projects [12, 15, 48]. Research consultants for CICO will (a) review main components of the interventions with the school’s CICO coordinator and data analyst and (b) plan ongoing implementation.

Coaching for CPP and CATS will consist of (a) goal setting [76], (b) self-reflection [77], and (c) performance feedback [78]. Participants will be told that they are expected to reach an 80% fidelity level when implementing the intervention. They will also learn that fidelity is set at 80% because the intervention would be more likely to be effective compared to a lower fidelity level [76]. Then, BHS will be asked to reflect on the previous session (e.g., *How do you think you did during the last CPP/CATS session or CICO case? What do you think went right? What do you think did not go well?*)*.* The consultant will provide BHS with approximate fidelity data for the previous CPP/CATS session or CICO case and note whether the fidelity threshold was achieved. Finally, the consultant will use audio clips from the previous session to encourage discussion about how the BHS handled student behavior in session, including the overall level of participation and enthusiasm, and disruptive or withdrawn behavior. The audio clips will be housed on a project website. Fidelity data will be provided to BHS regarding content fidelity (i.e., the material the BHS was supposed to cover in session). All consultation procedures will be detailed in a consultation manual. Coaching for CICO will consist of (a) providing performance feedback to the CICO coordinator and data analyst about their program (e.g., use of data to refer students to CICO, student progress monitoring) and (b) problem-solving implementation barriers.

### Data collection

Information about measures is shown in Table 1. The Tier 2 team will identify students for Tier 2 using the Strengths and Difficulties Questionnaire SDQ [46] completed by parents and teachers. As part of this process and depending on the need for services, the Tier 2 team might target certain grades/classes to screen students for mental health concerns. The cut-off score level for the SDQ is appropriate for identifying students at risk for a behavioral/mental health disorder [46].

During the training of BHS, we will collect data how many times during the training period BHS use the interventions that they have been trained on (adoption), and how many times and for how long video modules are accessed (training dosage). After post-intervention data are collected on students, BHS will participate in a survey and semi-structured interview to assess perceived acceptability (AIM) [40], feasibility (FIM) [40], appropriateness (IAM) [40], and usability [41] of the training and consultation procedures, and to gather their opinions about the support they received. Regarding the specific interventions, we will collect data on content fidelity [48] on an ongoing basis. Content fidelity is defined as the extent to which the prescribed components of the intervention are implemented.

We will also measure how many students are served per condition (penetration) [79], and pre- to post-changes in student mental health symptoms, as reported by parents (BASC-3) [52] and students (The Behavior and Feeling Survey-Youth Self Report) [80], and academic engagement (EvsD) [53], as reported by teachers.

#### Statistical analysis plan

The statistical analysis plan (SAP) will be updated and finalized before the data base lock. The SAP will provide comprehensive descriptive information of the statistical analysis plan, including approaches for summarizing primary and secondary endpoints at baseline and post-treatment. All statistical analyses will be performed using SAS® [81], version 9.4 or higher.

#### Data analyses by aim

Aim 1: To obtain input from school stakeholders about barriers and facilitators of remote online training by employing a user-centered research approach.

Research question # 1: What are the barriers to and facilitators of remote online training in participant schools?

We will import transcripts of semi-structured interview # 1 into NVivo for data management and analyses. Analyses will be guided by an integrated approach [82] that includes identification of a priori attributes of interest (i.e., constructs important to consider in the development of the remote training strategy), and modified grounded theory, which provides a rigorous, systematic approach to identifying emergent codes and themes.

Aim 2: To use user-centered design, guided by an iterative rapid prototyping approach, to develop asynchronous video modules based on preliminary studies and aim 1 data.

Hypothesis # 1: The final training video prototypes will be rated as feasible, acceptable, appropriate, and usable.

We will import transcripts of semi-structured interview # 2 into NVivo for data management and analyses. We will use mixed methods to integrate the quantitative and qualitative data. Consistent with Palinkas and colleagues [83], we will utilize the following design: the structure of the design is convergent (we will gather data from 5-point rating scales [AIM, IAM, FIM, usability] and qualitative data [i.e., semi-structured interviews, written answers] simultaneously and weigh them equally) and the function is of complementarity (to elaborate upon the quantitative findings to understand the *process* of implementation of remote consultation as experienced by stakeholders) [83, 84]. We will use the quantitative data to identify patterns in the qualitative data. To do this, we will enter quantitative findings into NVivo as attributes of each participant and these quantitative attributes will be used to categorize and compare important themes among subgroups.

Aim 3: To conduct a pilot trial of Remote Video vs. Remote Video plus Coaching.

The purpose of the pilot study is to examine “the real world” implementation of EBPs to students in the school setting. Our primary goal is to gather key measures to produce estimates related to implementation and student outcomes for the Remote Video condition and the Remote Video plus Consultation condition. Our research questions guiding our statistical analyses are:

Research question # 1: Will Tier 2 implementers assigned to Remote Video differ from those assigned to Remote Video plus Coaching on implementation outcomes (i.e., adoption, number of times and length of time accessing video modules; perceived feasibility, appropriateness, acceptability and usability of training strategy)?

Sub-aim 3a: To identify confounder variables associated with the two conditions regarding the use of the EBPs (CPP, CATS, CICO).

Research question # 2: Will students who receive Tier 2 support provided by Tier 2 BHS assigned to Remote Video differ from those assigned to Remote Video plus Coaching on student outcomes (i.e., penetration, mental health symptoms, academic engagement)?

Sub-aim 3b: To identify confounder variables associated with pre- to post-changes in student outcomes by the two conditions.

Sub-aim 3c: Estimate fidelity of CPP, CATS, and CICO by the two conditions.

Research question # 3: Will Remote Video plus Coaching be associated with higher fidelity compared to Remote Video?

The primary endpoints related to school staff implementing the interventions are measures of number of interventions per condition (adoption) [85], perceived feasibility of intervention (FIM) [40], intervention appropriateness (IAM) [40], acceptability of intervention (AIM) [40], usability [41], and intervention content fidelity [86]. Primary endpoints related to student outcomes are number of students eligible for interventions who use interventions, divided by the total number of students eligible for interventions (penetration) [79], and pre- to post-changes in student mental health symptoms (measured by BASC-3) [52], which include Aggression, Conduct Problems and Anxiety, and level of Academic engagement measured by the Behavioral Engagement, Emotional Engagement, Behavioral Disaffection and Emotional Disaffection subscales of EvsD [53].

Prior to the statistical comparison between groups (Remote Video and Remote Video plus Coaching), all pertinent variables collected for the pilot study will be presented as mean, standard deviation, median, minimum, maximum, and the 95% confidence intervals for continuous variables, while frequencies and proportions will be used for categorical variables. Presentation of summary statistics will be listed by study condition, geographic location, schools, and EBP (CPP, CATS, CICO). BHS and student characteristics (demographics and other potential confounders) will be compared between the two groups using the two independent samples *t*-test or the non-parametric Wilcoxon signed rank test to identify pre-treatment differences between the two groups. If the two groups are found to be statistically different in a pre-measured outcome, the pre-measurement(s) will be included in the subsequent analyses as a covariate using analysis of covariance (ANCOVA). The chi-squared test and Fisher’s exact test will be utilized for comparing the two conditions regarding categorical variables.

### Intent-to-treat analysis

Data will be analyzed using an intent-to-treat (ITT) approach, wherein each participant (BHS or student) will be kept in the arm to which the school was randomized, regardless of treatment received. In addition to creating pre/post-change scores and analyzing the data using *t*-tests (or the Wilcoxon signed rank test). For the purpose of generating statistical estimates for the anticipated larger scale study, we will explore the marginal models using the generalized estimating equations (GEE) [87, 88], for analyzing the pre- and post-repeated-measures endpoints related to student academic engagement and mental health symptoms. GEE will produce robust estimates that adjust for the clustering of students within schools. GEE will include study condition (Remote Video or Remote Video plus Coaching), time of measurement (pre/post), and arm × time interaction effects. This modeling approach will allow us to compare pre- to post-changes and the extent to which these changes differ across study arms. The nested nature of students within school/BHS will be explored by including schools as a covariate.

For the mixed-methods analyses for aim 3 (survey and semi-structured interview data about perceived appropriateness, feasibility, and acceptability of the training and consultation procedures, and BHS’s opinions about the support they received), we will use the same data analytic approach described in aims 1 and 2.

### Sample size considerations

This pilot study is designed to generate preliminary data to support a future larger scale hybrid type 3 study and is not powered to find statistically significant effect sizes. Based on our experience, a convenience sample size of 30 BHS will allow us to address aim 1 and aim 2. Forty-eight BHS and 209 students will participate in the pilot trial (aAim 3). The proposed pilot study aims to collect data and estimate effect sizes measuring the effect of Remote Video when compared to the Remote Video plus Coaching. Based on data obtained from our recently completely NIH-funded study [12, 48], we estimated that 24 BHS in the Remote Video condition and 24 BHS in the Remote Video plus Coaching condition produce a two-sided 95% confidence interval (95% CL) in mean differences in content fidelity equal to a mean difference ± 4.7, assuming that the estimated standard deviation for each condition is equal to 16. We anticipate that a total of 178 evaluable students will participate in the study, 89 students in Remote Video and 89 students in Remote Video plus Coaching. A two-sided 95% confidence interval for mean differences in pre-post changes in student mental health symptoms and academic engagement between the two conditions will be estimated as mean difference ± 1.2 4. We assumed that the estimated standard deviation in each condition will be 8. Sample size justification was reported using PASS 13 software [89].

## Discussion

There are relatively few studies that evaluate online delivery of training in mental health. The prior research on online approaches for training and consultation with community providers has primarily been conducted in the context of large implementation trials [35]. This methodology typically precludes random assignment to condition and limits opportunities to develop training programs that fit into the existing context. An interactive process involving the user-centered design can increase buy-in, and enhance the fit, sustainability, and effectiveness of a training program for underserved populations [90, 91]. And, although research exists on remote training of non-specialist staff in traditional mental health and medical settings [92], few studies have systematically evaluated remote mental health training of school-based mental health staff. Additionally, a significant shortcoming is that most remote training studies have not attended to implementation issues and typically have not included an implementation framework [93]. In one review of the literature, only 5% of remote training studies mentioned any theoretical approach to implementation [94]. There is a clear need for well-designed remote training studies focused on training in non-traditional settings.

### Innovation

Our study is innovative in four areas:

- It incorporates mental health EBPs into an existing school-wide service delivery approach in rural schools, thereby improving feasibility. This is very innovative in the rural school context;
- It develops a remote training strategy using a collaborative, iterative approach (*user-centered design and rapid prototyping*), increasing both feasibility and buy-in;
- It employs “gold-standard” training methods, which should lead to better child outcomes; and
- To our knowledge, this is the first study that proposes to test the efficacy of two remote training strategies for mental health in rural schools.

### Scientific rigor and reproducibility

We use rigorous methods to compare outcomes, using measures with strong psychometric properties, multiple data collection strategies (surveys, interviews, independent coding), quantitative and qualitative data, and sound analytical methods. All phases of the study are carefully described in order to enable replication of methods [95, 96].

### Public health impact

Given the lack of well-trained mental health professionals in rural settings and the stark disparities in access to services, the development and pilot-testing of remote training strategies for BHS in under-served rural schools could lead to significant public health impact. We believe that this study will make significant contributions to the fields of school mental health, and services and implementation research in rural areas.

### Potential problems and alternate solutions

Some school personnel might not be able to handle the expectations placed on them with regard to study participation. For example, some school staff might not be able to keep up with uploading audio-recordings from the student intervention sessions so that consultants can review the recordings in time for the next consultation session. Our research team has successfully obtained this type of data in previous studies; we will monitor this closely and provide support as needed.

The turnover rate among teaching staff and principals is relatively high in rural schools. This could affect the work of the Tier 2 implementers. However, most turnover takes place during the summer months and not during the academic year. As such, we will be able to address this problem by thoroughly training new school personnel at the beginning of each academic year and providing consultation support according to the training manual.

There could be a lag in the identification of students for Tier 2 or in obtaining parent consent to let the students participate in a Tier 2 group. We will work closely with the Tier 2 BHS to identify students for Tier 2. We will remind Tier 2 BHS to get parents to complete measures and to get parent permissions for members of the research team to contact them in order to seek informed consent.

It could be a challenge to enroll students and collect measures remotely. We will work closely with the schools if we encounter problems in this area.

### Limitations

The current study will not be able to obtain implementation or effectiveness data on students who need individualized supports (Tier 3). Collecting these types of data would be beyond the scope of the current study. Results may not generalize to non-rural schools because of the unique characteristics (e.g., remote physical location, limited resources) of rural schools. However, results should generalize to any rural school district in the country.

## Data Availability

Not applicable.
